# Face Recognition in SSPP Problem Using Face Relighting Based on Coupled Bilinear Model

**DOI:** 10.3390/s19010043

**Published:** 2018-12-22

**Authors:** Sang-Il Choi, Yonggeol Lee, Minsik Lee

**Affiliations:** 1 Department of Computer Science and Engineering, Dankook University, 126, Jukjeon-dong, Suji-gu, Yongin-si, Gyeonggi-do 448-701, Korea; choisi@dankook.ac.kr; 2 Police Science Institute, 100-50, Hwangsan-gil, Sinchang-myeon, Asan-si, Chungcheongnam-do 31539, Korea; pattern@police.go.kr; 3 Division of Electrical Engineering, Hanyang University, 55 Hanyangdaehak-ro, Sangnok-gu, Ansan-si, Gyeonggi-do 15588, Korea

**Keywords:** single sample per person problem, face relighting, coupled bilinear model

## Abstract

There have been decades of research on face recognition, and the performance of many state-of-the-art face recognition algorithms under well-conditioned environments has become saturated. Accordingly, recent research efforts have focused on difficult but practical challenges. One such issue is the single sample per person (SSPP) problem, i.e., the case where only one training image of each person. While this problem is challenging because it is difficult to establish the within-class variation, working toward its solution is very practical because often only a few images of a person are available. To address the SSPP problem, we propose an efficient coupled bilinear model that generates virtual images under various illuminations using a single input image. The proposed model is inspired by the knowledge that the illuminance of an image is not sensitive to the poor quality of a subspace-based model, and it has a strong correlation to the image itself. Accordingly, a coupled bilinear model was constructed that retrieves the illuminance information from an input image. This information is then combined with the input image to estimate the texture information, from which we can generate virtual illumination conditions. The proposed method can instantly generate numerous virtual images of good quality, and these images can then be utilized to train the feature space for resolving SSPP problems. Experimental results show that the proposed method outperforms the existing algorithms.

## 1. Introduction

For the last 30 years, researchers have investigated issues associated with finding and developing effective methods of face recognition [1,2]. From the results of such efforts, diverse methods of face recognition have been developed, giving users a certain level of face recognition performance under both controlled and uncontrolled environments. However, many issues are yet to be resolved in order to expand the range of effective applications of face recognition. Basically, the difficulty in face recognition is caused by intrinsic factors, such as varied expressions, aging, makeup, or worn accessories, and extrinsic factors, such as varied poses or illumination that could change the facial image to resemble various other facial images.

The methods that have been developed and employed for face recognition thus far can be divided into two types: those that use three-dimensional (3D) face models [3,4] and those that use the features from two-dimensional (2D) facial images [5,6]. However, obtaining a 3D face model requires special equipments or needs numerous computational resources; limitations are therefore unavoidable for instruments with small-scaled computational resources, such as mobile devices or for other generally employed equipments [7].

The appearance-based methods that use 2D images represent images as a 2D array or as 2D vectors and recognize facial images using statistical information on the pixel density [8,9,10,11]. These methods commonly define diverse objective functions and covariance matrices to extract features available for the recognition of faces. Appearance-based methods exploit supervised [9], semi-supervised [11], or unsupervised learning [10]. Among them, the strategy used in discriminant-analysis-based methods involves reducing the distribution of within-class images. Moving away from the distribution of different class images is very effective in an ideal case of small within-class variance but large between-class variance [9,12]. In addition, appearance-based methods can construct compact, low-dimensional spaces which retain the intrinsic characteristics of the original face [13]. Since the low-dimensional feature vector is used as an input of the classifier, enabling real-time operation with less computational resources, these methods can easily be applied to diverse applications, including mobile devices.

Securing several images of a person’s face is very important to enable the favorable face recognition performance of appearance-based methods. Since the facial image is generally converted into high-dimensional data, the dimension of the input (image) space is frequently higher than the number of available image samples. In such cases, the within-class covariance matrix becomes singular and thereby restricts various types of discriminant analyses. This problem is referred to as the small sample size (SSS) problem [14]. Although several methods, such as Fisherface [8] and Discriminant Common Vector (DCV) [9], have been presented to solve SSS problems, the number of training samples needed for one person still affects the performance of such appearance-based methods. The image acquisition environment can be diversified in a practical face recognition system and, owing to such environmental variation, the images of the same person can appear to have diverse features. Therefore, in the case of a small number of image samples, the estimation of within-class variance becomes inaccurate and consequently degrades the generalization performance of the face recognition system [15].

In particular, as an extreme case of the SSS problem, the case of only one image per person is called the single sample per person (SSPP) problem [13,16,17,18]. The SSPP problem often cannot be resolved with the methods used to solve general SSS problems because the variance of images within the same class cannot be defined. In addition, in many cases, it is difficult to obtain several face images of one person for applications requiring face recognition [16]. More specifically, most large-scale identification applications for the purposes of law enforcement, driver’s license, or passport-photo identification generally save only one image for each person in each database; thus, the task of securing more than a single image of one person is difficult to perform due to the increasing cost and time involved, along with the increasing number of people or objects that need to be recognized. In addition, the collected images should be taken under various conditions in order to encompass different variations of faces to effectively improve the performance of face recognition [13,16].

On the other hand, saving only one image of a person can be a significant advantage for real-world applications if SSPP problems can be solved easily. This can reduce the practical cost of collecting the training samples required to establish face recognition systems, together with the storage cost of the database. Accordingly, the scope of the application of face recognition systems can be expanded, including the surveillance of public spaces of railway stations or airports, etc. [16,19]. Thus, the resolution of SSPP problems is important with respect to the performance of face recognition, as well as the utility of the system.

Numerous methods have been suggested to solve the problems arising from having too few training images for each person. The methods proposed in [20,21,22] use new representation schemes to extract more information from a single image. In [20], images were derived from the original image by perturbing the face matrix’s singular values. Therefore, the derived images and original images supplemented the lack of training images. In the ${E(\mathrm{PC})}^{2}A$ of [21], the original image and its corresponding half-, first-, and second-order projected images were used to enlarge the training set. In [22], new samples were synthesized based on the interclass relationship by using the weighted combination of pairs of original images. However, the images created through the above methods were highly correlated, and, thus, it is difficult for the system to recognize them as independent training images [23].

In other methods proposed to solve SSPP or SSS, virtual images are created through geometrical transformation, such as the rotation or bilateral symmetric transformation of the original images. In [24], virtual images were generated using a symmetry transform and linear combination of the interclass. The method in [25] exploited the symmetrical structure of a face to generate new training samples. However, these methods focus mainly on an arithmetic increase of the training set and do not properly reflect the diversity of facial images, which vary according to environmental changes, such as illumination, etc.

Sparse representation methods [1,26,27] can also be utilized to solve the SSPP problem. Sparse representation-based classification (SRC) [26] represents a test image as a linear combination of training images. However, since it does not consider the case of having one image per person [27], the performance has been found insufficient for managing the SSPP problem. Extended SRC (ESRC) [27] learns the auxiliary intraclass variant dictionary and uses the undersampled training images to handle the intra- and interclass variations. Although ESRC improves the robustness of face recognition, it necessitates collecting images that contain certain variations of the same person. Gao et al. [28] proposed semi-supervised sparse representation to solve face recognition when there are only a few labeled samples.

Recently, there have been more proposals with the aim to solve the SSPP problem. Wei and Wang [29] proposed robust auxiliary dictionary learning to solve the SSPP problem. They utilized additional samples that are not relevant to the recognition problem to supplement the training data and handle unwanted sample corruptions, such as occlusions. Ma et al. [30] proposed a non-rigid face registration method via a regularized Gaussian field criterion to reduce the non-rigid geometric variations in face images. Ding and Tao [31] proposed the Trunk-Branch Ensemble convolutional neural network (CNN) model to retrieve a blur-free face image from video data, which can be efficiently used in face recognition. Masi et al. [32] used various facial image synthesis techniques to account for pose, shape, and expression variations when training a CNN model for face recognition. There have also been some proposed one-shot- or few-shot-learning methods based on CNN models [33,34]. Wang et al. [33] proposed the balancing regularizer and shifting center regeneration for efficient one-shot learning for face recognition. Wu et al. [34] proposed the use of a hybrid classifier, comprising CNN and nearest neighbor, for low-shot-learning-based face recognition.

There are many factors to consider in the SSPP problem, such as illumination, pose, occlusion, and so forth. Among them, this paper focuses on addressing variations in illumination, i.e., we explore the possibility of synthesizing virtual images from a single image with various realistic illuminations while considering quality, efficiency, and practicality. There have been some approaches to the synthesis of illumination for face recognition [13,35]. For example, a recent SSPP algorithm roughly mimicked illumination variations to improve recognition performance [13]. In the algorithm, facial images under various illumination conditions were created from bidirectional integral features captured under lighting with different orientations. However, the method has a limitation whereby the variations in illumination in vertical or arbitrary directions, except for lateral moves, are difficult to manage. Another possibility is using GAN [36] to generate synthetic images, and there have been some successes reported for this task in the recent literature. It is true that GAN can be a viable path; however, it comes with prices of its own, e.g., it requires a lot of data to train it, a lot of efforts to stabilize the training procedure, etc.

On the other hand, we may grasp the possibility of using existing face relighting methods for the SSPP problem, because face relighting itself has been an active area of research for some time. In some approaches [37,38], the morphable model [39] is modified to handle illumination changes. These models are fit to the input image to acquire the 3D shape and illumination, and then the illumination parameter is replaced to synthesize a novel relit image. These methods tend to give realistic results due to the power of the morphable model; however, they require a significant amount of computing power. Another common approach is to utilize 2D illumination models, in which texture information may be included, that are trained from exemplars [40,41,42,43]. These 2D methods approximate illumination changes based on some representations, such as spherical harmonics [44], and they are often represented by linear subspaces. These methods are much simpler than those using morphable model approaches and are less computationally demanding. Although they lack the estimation of accurate shape information, this can be efficiently counterbalanced (in real time) by normalizing 2D geometric variations based on recently developed facial feature localization methods [45]. However, the image qualities of these 2D methods have been usually very poor (i.e., mostly blurry) due to the limitations of linear subspaces, which were later addressed in patch-based approaches [46,47] with increased complexities. Other than these, some methods have been proposed that utilize only a single 3D reference [48] or a logarithmic total variation model [49] to generate rough relit images, although the qualities of their results are poor.

Overall, there is a trade-off between the computational cost and the quality of virtual images and, accordingly, the recognition performance. These days, face recognition is being realized in various mobile devices for security purposes, so computationally heavy algorithms are not adequate for practical situations, but, at the same time, performance should be guaranteed to ensure reliability. In order to reduce the gap between these two conflicting goals, in this paper, we propose a novel face relighting method for SSPP problems which instantly synthesizes various relit images with good qualities. Our proposed model is based on some observations. It has been long observed in the literature that high-frequency components in an image are related more to the reflectance (or texture) of an object than to the illuminance. On the other hand, it has been also shown in the literature that illumination components can be effectively approximated by up to 99% of the total energy by a low-dimensional subspace [44]. Hence, using a 2D subspace model only for the illumination components can be a good alternative for generating virtual images. However, in this case, we need to estimate a dense reflectance map during the illumination synthesis process, which can overcomplicate the algorithm. In [50], on the other hand, it was shown that facial features extracted from an image strongly correlate to its 3D shape. A subspace model for facial images, even one that produces blurry images from image synthesis, is effective for estimating the ‘state’ of the images, such as light conditions and facial attributes, and the method in [50] utilizes this fact to bridge between the input image and the output 3D shape. This suggests that, since a 3D shape strongly affects its illuminance, facial illuminance can also strongly correlate with the corresponding facial features. In fact, we can expect an even stronger correlation between the illuminance and the facial features, because illuminance is qualitatively more similar to an image than a 3D shape.

Therefore, in this study, we built a coupled model of image bases (including texture) and illumination bases (without texture). While facial image bases (including texture) yield poor qualities in image synthesis, they can provide fairly good information on light conditions and facial attributes. This information can be combined with the illumination bases to produce virtual illuminance (without texture), which is relatively insensitive to poor resolution, and they can be used afterward to efficiently estimate a dense texture map (or reflectance map) of the input image. In this way, we can synthesize a realistic image without the need for any complicated 3D fitting procedure.

The proposed method constructs a feature space based on these synthesized images to recognize a face image in order to secure robust face recognition performance and to solve the problems of SSPP. The experimental results show that the proposed method improves face recognition performance with a single training sample per person, even in the presence of illumination variation. The proposed virtual image synthesis method can also be used in deep learning for data augmentation. In order to fully handle the SSPP problem, there are many factors, such as pose, occlusion, makeup, facial variation, as well as illumination, that have to be considered. However, solving all of these components with a single algorithm is very challenging, so we left it as future work.

The remaining content of this paper includes the following sections. Section 2 presents the proposed coupled bilinear model, which is used to create virtual images under various light conditions. Then, we describe the construction of the feature space based on the synthesized images for robust face recognition under environmental variations. In Section 3, the face recognition performance of the proposed method is evaluated on various face databases. Section 4 then provides the conclusions of this study.

## 2. Proposed Method

### 2.1. Training Coupled Bilinear Model

**Figure 1:**
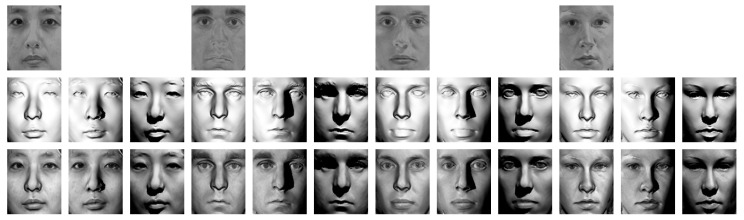
Examples of reflectance and illuminance of face images (synthesized from the FRGC 2.0 database [51]): (**Top**) Reflectance (or albedo); (**middle**) illuminance; and (**bottom**) corresponding images.

The goal of the paper is to solve the SSPP problem in face recognition based on the efficient generation of virtual images for novel illumination conditions. First, we define some terms that are used in the rest of the paper. The term “image” represents the actual image of a human face for a certain person under a certain light condition. On the other hand, “illuminance” represents the brightness of the corresponding image, excluding the effect of reflectance or albedo, which roughly represents the natural color of the surface for each pixel. This concept is depicted in Figure 1. To generate a virtual image with a novel light condition, first we have to separate the illuminance and the reflectance from the input image, and then we synthesize adequate illuminance for the new light condition. In this work, this procedure is performed based on a pre-trained coupled model of facial image and illuminance. The proposed relighting method utilizes this coupled model to efficiently retrieve the illuminance from a given face image. The overall procedure for training the coupled model and generating virtual images is summarized in Algorithms 1 and 2, respectively.

The coupled model consists of two bilinear models: the image model and the illumination model. Each model is represented by bilinear bases in order to handle both lighting variations and facial attributes, both of which are variables for the bilinear models that are explained later. These bilinear bases can be described using tensors; before proceeding further, we briefly introduce two basic tensor operations. (For more details on tensor algebra, please refer to [52].) A tensor is a multidimensional extension of vectors (first-order tensors) and matrices (second-order tensors). Let $A\in R^{n_{1}\times n_{2}\times \cdots \times n_{N}}$ be an *N*th-order tensor and $M\in R^{n_{k}^{\prime }\times n_{k}}$ be a matrix. Then, the mode-*k* product, a generalization of the matrix-vector product, of these can be expressed as

(1) $$B=A\times_{k}M,$$

where an element of $B\in R^{n_{1}\times n_{2}\times \cdots \times n_{k}^{\prime }\times \cdots \times n_{N}}$ is defined as

(2) $$B_{i_{1}i_{2}\cdots i_{k}^{\prime }\cdots i_{N}}=\sum_{i_{k}}A_{i_{1}i_{2}\cdots i_{k}\cdots i_{N}}M_{i_{k}^{\prime }i_{k}}.$$

The *N*-mode singular value decomposition (SVD) [52], or the Tucker decomposition, is a generalization of the SVD for tensors. For example, A can be decomposed as

(3) $$A=C\times_{1}U_{1}\times_{2}U_{2}\times_{3}\cdots \times_{N}U_{N},$$

where C is a (*N*th-order) core tensor and $U_{i}$ is an orthogonal mode matrix. Note that the core tensor does not necessarily have a diagonal structure, but its elements usually have much higher magnitudes for lower indices, like the singular values in SVD.

Here, we adopt a popular assumption about images, i.e., an image is a combination of illuminance and reflectance. Let $L\in R^{n_{y}\times n_{x}}$ be the illuminance of an image (size of $n_{y}\times n_{x}$) and $T\in R^{n_{y}\times n_{x}}$ be the texture (i.e., reflectance). Then, the image Ψ can be represented as Ψ≜T⊙L, where ⊙ is the Hadamard product or the element-wise product. Accordingly, the illumination and image models used in this paper are the models for L and Ψ, respectively. Both models have two variables: the light condition and the facial attributes. The light condition variable, which is denoted by the symbol l, determines the light condition. Changing the values of l will change the light condition of the resulting image or illuminance without changing the identity of the person. On the other hand, the facial attributes, denoted by ϕ, represent the characteristics that are different for each person. Hence, modifying ϕ will change the personal identity of the resulting image or illuminance. These variables are feature vectors (as those in feature extraction techniques) and are not explicitly represented in a physically meaningful way.

First, the image model can be described as

(4) $$\Psi \left(\phi_{\Psi },l_{\Psi }\right)=\left(M_{\Psi }+H_{\Psi }\times_{4}\phi_{\Psi }^{T}\right)\times_{3}l_{\Psi }^{T},$$

where $l_{\Psi }\in R^{n_{l}}$ and $\phi_{\Psi }\in R^{n_{l}}$ are the light condition vector and the facial attribute vector, respectively. $M_{\Psi }\in R^{n_{y}\times n_{x}\times n_{l}}$ is the *mean* tensor, which can be regarded as the model of the average face for various light conditions, and $H_{\Psi }\in R^{n_{y}\times n_{x}\times n_{l}\times n_{\phi }}$ is a quartix (fourth-order tensor) that contains the bilinear bases. This kind of model has been often used for face images in the literature [50], but what is different here is that we define another model for illuminance: the illumination model. This model is defined similarly as

(5) $$L\left(\phi_{L},l_{L}\right)=\left(M_{L}+H_{L}\times_{4}\phi_{L}^{T}\right)\times_{3}l_{L}^{T}.$$

Note that we deliberately avoid defining an explicit model for T in this setting. This is because T is likely to be sensitive to the resolution of the model, i.e., T represented by a subspace model can be blurry, unlike L, which is more robust to poor resolutions. As explained in Section 1, our goal is to retrieve the less-sensitive illuminance information rapidly and to calculate the texture based on this information robustly. The image model is used to extract the information about the light condition and the facial attributes effectively: if we only have the illumination model, then T needs to be explicitly estimated along with other parameters, which will complicate the algorithm.

**Algorithm 1** Training the coupled bilinear model.
1:Given training image data $\Psi^{\prime }$ and its corresponding illuminance data;2:Calculate $M_{\Psi }$, $H_{\Psi }$, $M_{L}$, and $H_{L}$ based on Equations (6) and (7).3:Calculate $W_{\Psi }$ based on Equation (8).4:Calculate $P_{\phi }$ and $P_{l}$ based on Equation (9).


**Algorithm 2** Synthesizing virtual images.
1:Given an input image Q and novel light condition vectors $l_{L,i}$’s;2:Retrieve $\phi_{\Psi }$ and $l_{\Psi }$ from Q based on Equation (10).3:Convert $\phi_{\Psi }$ and $l_{\Psi }$ to $\phi_{L}$ and $l_{L}$, respectively, based on Equation (11).4:Retrieve $T_{Q}$ based on Equation (12).5:Synthesize virtual images as $Q_{i}=T_{Q}⊙L\left(\phi_{L},l_{L,i}\right)$.


The above models can be trained based on exemplars synthesized from a 3D face database. In this study, we used the FRGC 2.0 database [51] to train the models. $m_{\phi }=500$ 3D shapes were selected and preprocessed, similar to the approach used in [53], and were subjected to $m_{l}=100$ evenly distributed artificial point light sources to collect the training samples for both models, which were then resized to 120×100. Finally, *N*-mode SVD was applied to the training samples and unimportant dimensions were truncated to yield

(6) $$\Psi^{\prime }-M_{\Psi }^{\prime }\times_{4}1\approx G_{\Psi }\times_{1}U_{y}\times_{2}U_{x}\times_{3}U_{l}\times_{4}U_{\phi },$$

where $\Psi^{\prime }\in R^{n_{y}\times n_{x}\times m_{l}\times m_{\phi }}$ is a quartix consisting of training image samples of various light conditions and faces, and $M_{\Psi }^{\prime }\in R^{n_{y}\times n_{x}\times m_{l}}$ is the average of $\Psi^{\prime }$ along the fourth mode. $G_{\Psi }$ is the truncated core tensor, and $U_{i}$ is the *i*th truncated mode matrix. Based on this result, the terms in Equation (4) can be calculated as

(7) $$\begin{array}{cc} H_{\Psi } & =G_{\Psi }\times_{1}U_{y}\times_{2}U_{x},\\ M_{\Psi } & =M_{\Psi }^{\prime }\times_{3}U_{l}^{T}. \end{array}$$

The same procedure was applied to the illuminance samples to yield the illumination model (Equation 5). The illuminance samples were created by excluding the textures during the same synthesis procedure. The sizes of the core tensors were 40×30×10×150 for the image model and 40×30×100×150 for the illumination model. Note that, in this work, the images were rendered based on the Lambertian assumption, and cast shadows were also rendered based on the underlying 3D shapes, although it is possible to use more complicated lighting models. To normalize the geometric variations, recently developed facial feature localization methods [45] can be used, but, in this study, normalizing based on eye and mouth coordinates was sufficient for the performance of the proposed SSPP method.

Much evidence in the literature implies that the above two models likely have a strong correlation with each other [50]. Hence, we can define the relationships between the parameters of these two models, i.e., $\phi_{\Psi }$ to $\phi_{L}$ and $l_{\Psi }$ to $l_{L}$. Before finding the relationships, we extract only the components that have strong correlations in the parameters, because there may be some noise in the models, based on the canonical correlation analysis (CCA) [54]. The CCA problem for $\phi_{\Psi }$ and $\phi_{L}$ can be defined as

(8) $$\begin{array}{cc} \max_{w_{\Psi },w_{L}}\; & w_{\Psi }^{T}C_{\Psi L}w_{L},\\ s.t.\; & w_{\Psi }^{T}C_{\Psi }w_{\Psi }=w_{L}^{T}C_{L}w_{L}=1, \end{array}$$

where $C_{\Psi L}$ is the cross-covariance of $\phi_{\Psi }$ and $\phi_{L}$, while $C_{\Psi }$ and $C_{L}$ are the covariances of $\phi_{\Psi }$ and $\phi_{L}$, respectively. This problem can be efficiently solved by the generalized eigenvalue decomposition. In this way, we can find those components with the strongest correlations.

Based on the strongly correlated components extracted by the CCA, we find a linear mapping to convert a given $\phi_{\Psi }$ to the corresponding $\phi_{L}$. First, we project all the $\phi_{\Psi }$ of the training samples to the CCA components to extract only the strongest components, and we then find a direct linear mapping from the projected samples to the corresponding $\phi_{L}$. Let $W_{\Psi }$ be a matrix composed of different vectors of $w_{\Psi }$ (100 components were extracted for the proposed method); then, linear mapping from $\phi_{\Psi }$ to $\phi_{L}$ can be found as $P_{\phi }=C_{\Psi L}W_{\Psi }^{T}$, which is the least-squares estimate of

(9) $$\min_{P}\;E\;\left[{∥\phi_{L}-PW_{\Psi }^{T}\phi_{\Psi }∥}^{2}\right],$$

based on the knowledge that $W_{\Psi }^{T}C_{\Psi }W_{\Psi }=I$. This $P_{\phi }$ will be used later in the virtual image synthesis to convert $\phi_{\Psi }$ of a given test image to the corresponding $\phi_{L}$.

Although we could also apply the CCA to $l_{\Psi }$ and $l_{L}$, this would not be useful because the dimension of $l_{\Psi }$ is small (value of 10) in our algorithm. Hence, we directly find the linear mapping $P_{l}$ based on least squares. Note that the samples of $\phi_{\Psi }$, $\phi_{L}$, $l_{\Psi }$, and $l_{L}$ for these procedures can be found during the *N*-mode SVD steps of both models.

### 2.2. Synthesizing Novel Illuminations

Based on the coupled model, we can synthesize novel illuminations for a new input image. First, we need to extract the ‘state’ of the image. This can be achieved by fitting the image model to the image:

(10) $$\min_{\phi_{\Psi },l_{\Psi }}\;{∥Q-\left(M_{\Psi }+H_{\Psi }\times_{4}\phi_{\Psi }^{T}\right)\times_{3}l_{\Psi }^{T}∥}^{2}+\lambda {∥\phi_{\Psi }∥}^{2},$$

where Q is the input image. Here, a ridge regularizer is introduced for robustness. This regularization can also ensure that the model is working in the ‘effective’ region [50]. This problem can be solved by alternating least squares (ALS) as for any other bilinear model [50]. Even though this is a non-convex problem that requires iterations, in [50], a method was provided to reduce the computation dramatically by utilizing the mode matrices $U_{y}$ and $U_{x}$, which makes it possible to estimate $\phi_{\Psi }$ and $l_{\Psi }$ instantly.

After finding the solutions of $\phi_{\Psi }$ and $l_{\Psi }$, they are translated to the parameters of the illumination model as

(11) $$\begin{array}{cc} \phi_{L} & =P_{\phi }\phi_{\Psi },\\ l_{L} & =P_{l}l_{\Psi }. \end{array}$$

Then, the illuminance $L_{Q}$ of the input image can be estimated by Equation (5).

**Figure 2:**
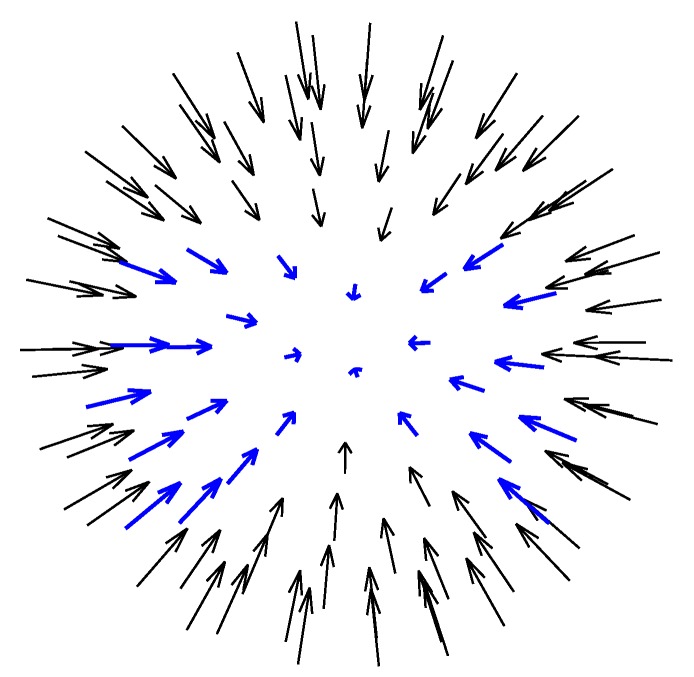
One-hundred light conditions. Synthesized images in the directions indicated by blue arrows are used in the proposed face recognition system.

Now, the texture $T_{Q}$ of the input image can be found by the relation $Q=T_{Q}⊙L_{Q}$; however, this incurs a problem: as pointed out in [37], finding $T_{Q}$ based on this equation can be ill-posed if the light condition in the image is extreme (i.e., contains large shadows), as this results in division-by-zero problems. To handle this robustly, in this work, we utilized the image model to compensate for the shadow area. At this point, we define the canonical light conditions, $l_{\Psi ,i}$ (for the image model) and $l_{L,i}$ (for the illumination model), which represent a set of standard light conditions. In this study, the 100 light conditions used for training the coupled model are reused as the canonical light conditions. Then, by supplementing information from the image model for these canonical conditions, we can have a robust estimate of $T_{Q}$:

(12) $$\begin{array}{cc} \min_{T}\; & {∥Q-T⊙L_{Q}∥}^{2}+\\ & \frac{\gamma }{m_{l}}\sum_{i}{∥\Psi \left(\phi_{\Psi },l_{\Psi ,i}\right)-T⊙L\left(\phi_{L},l_{L,i}\right)∥}^{2}, \end{array}$$

where $m_{l}$ is the number of canonical light conditions, and γ>0 is a constant. This least-squares problem obviously has a closed-form solution. Note that since this formulation does not have a matrix multiplication but only has element-wise multiplications, the solution can be calculated separately for each pixel. After finding $T_{Q}$, the *i*th relit image $Q_{i}$ can be synthesized as $Q_{i}=T_{Q}⊙L\left(\phi_{L},l_{L,i}\right)$, which can be used as a virtual image for robust SSPP recognition. Figure 2 shows the directions corresponding to the 100 canonical light conditions.

### 2.3. Face Recognition Using Synthesized Images

To construct a feature space for face recognition, the DCV method was selected among the appearance-based methods for this study. DCV, which is a representative supervised method based on discriminant analysis, demonstrates a favorable performance in the classification of high-order data containing abundant null space, such as face images [9,12]. (Feature extraction methods other than DCV can also be employed for this purpose; DCV was selected in this study for convenience). Let the *k*th image of the *c*th class be $x_{k}^{c}$, and let $\mu_{c}$ and μ be the average of all images belonging to the *c*th class and the average of whole images, respectively. Most appearance-based methods, including DCV, define a within-class scatter matrix ($S_{W}$) and between-scatter matrix ($S_{B}$) for the discriminant analysis. A discriminative feature space is then constructed with the projection matrix (*W*), which comprises projection vectors (w), that satisfies the predefined objective function.

(13) $$\begin{array}{cc} S_{B} & =\frac{1}{K}\sum_{i=1}^{c}K_{i}\left(\mu_{i}-\mu \right){\left(\mu_{i}-\mu \right)}^{T},\\ S_{W} & =\sum_{i=1}^{c}\sum_{x_{k}^{c}\in \left\{c_{i}\right\}}\left(x_{k}^{c}-\mu_{i}\right){\left(x_{k}-\mu_{i}\right)}^{T}. \end{array}$$

For the case of the DCV method, since the null space of $S_{W}$ contains sufficient discriminative information, it finds the projection matrix $W_{DCV}$ that satisfies the following objective function in the space, where $|W^{T}S_{W}W|=0$ and $|W^{T}S_{B}W|\neq 0$.

(14) $$\begin{array}{c} W_{DCV}=\underset{|W^{T}S_{W}W|=0}{\arg\max}\left|W^{T}S_{B}W\right|. \end{array}$$

By using $W_{DCV}$, an image sample $x_{k}^{c}$ is represented as a feature vector $y_{k}^{c}=W_{DCV}^{T}x_{k}^{c}$ in the DCV feature space.

However, despite the excellent discriminative power of the null space of $S_{W}$, methods such as DCV cannot be used in problems of SSPP that have only one image per person, because $S_{W}$ cannot be defined. In addition, since information about the variation in a face caused by environmental change is unavailable, the within-class variation cannot be reflected in the training process. As a solution to such problems, the proposed method improves the feasibility of appearance-based face recognition methods by synthesizing several images from one image, and it enhances the generalization performance of face recognition to handle the environmental changes by modeling the within-class variation.

If the synthesized images are to be effective in constructing the robust face recognition system to cope with environmental changes, the changes in facial appearance attributable to illumination conditions should be reflected in the synthesized images. In order to arrange the synthesized images for use in constructing the feature space for face recognition, we compared the distribution of the images synthesized from 100 light conditions (Figure 2) in the DCV feature space with that of real images captured in various illumination conditions.

**Figure 3:**
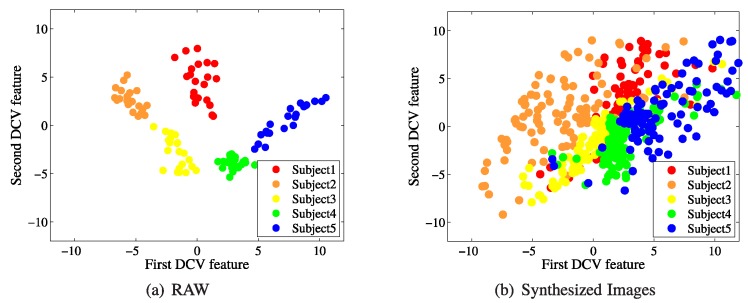
Distributions of samples for five subjects in the two-dimensional Discriminative Common Vector (DCV) feature space: (**a**) real images; and (**b**) synthesized images (proposed method).

Figure 3 shows the distribution of images from the CMU database, which is widely used in face recognition studies that deal with illumination variation. The CMU database contains face images of 65 subjects captured under 21 different illumination conditions. Among them, 7 images of 40 subjects, who were imaged under different illumination conditions, were used to construct the 39-dimensional DCV feature space. Then, for the remaining 25 subjects, the 21 real images captured from different light conditions and the 100 images which were synthesized, using the proposed method, from one image taken under frontal lighting were projected to the DCV feature space.

Figure 3a,b show the projection of 105 real images (5 subjects × 21 images) and 500 images (5 subjects × 100 images), which were synthesized by using the proposed method on 5 subjects among all 25 subjects into a 2D DCV feature space. In Figure 3a, for the case of real images from the CMU-PIE database, the images of each subject captured in different illumination conditions were formed into clusters, and each cluster was distributed separately in the feature space. Five-hundred synthesized images in Figure 3b were also clustered together for each subject; however, clusters partially overlap with each other due to the greater variance of each cluster. Thus, in this study, some of the illumination conditions among all 100 light conditions were limitedly selected based on the distribution of real images, and their corresponding synthesized images were used to construct an effective feature space for the face recognition system.

Let the index for light condition and the DCV feature vector for the synthesized image of the *c*th subject be l(l=1,…,100) and $y{\left(syn\right)}_{l}^{c}$, respectively. First, we calculated the standard deviations of $y{\left(syn\right)}_{l}^{c}$ for the real images of each subject ($\sigma_{c},c=1,\ldots ,25$) and the maximum value $\sigma_{max}$ among them. Then, each $y{\left(syn\right)}_{l}^{c}$, which is within a distance of $\kappa \cdot \sigma_{max}$ from the corresponding mean ($m_{syn}^{c}$) of the synthesized image for each subject, was distinguished as a set $Candi^{c}$.

(15) $$Candi^{c}{=\{y\left(syn\right)}_{l}^{c}{|∥y\left(syn\right)}_{l}^{c}-m_{syn}^{c}{∥}_{2}<\kappa \cdot \sigma_{max}\}.$$

The light condition indices in $Candi^{c}$ differ for each subject *c*. Thus, we selected 26 light conditions, all of which were included in each $Candi^{c}$, for the construction of the feature space for face recognition. The selected light conditions are marked in blue in Figure 2. The images under these light directions contain the most representative facial appearances under light variations; on the other hand, those under the other light directions, which have large areas of shadows, provide relatively little information about the person.

**Figure 4:**
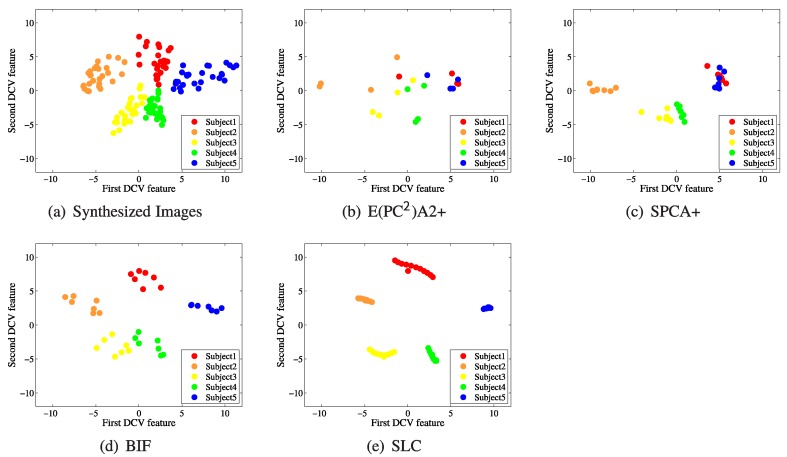
Distributions of image samples that were generated from a single image in the two-dimensional DCV (Discriminative Common Vector) feature space: (**a**) synthesized images (proposed method); (**b**) E(${\mathrm{PC}}^{2}$)A2+; (**c**) SPCA+; (**d**) BIF; and (**e**) SLC.

Figure 4 shows the distributions of five subjects that were generated from a single image using several methods addressing with SSPP problems: E(${\mathrm{PC}}^{2}$)A2+ [21], SPCA+ [20], BIF [13], and SLC [24]. After generating images from a single image taken in the normal condition (frontal illumination) using several methods, we plotted the image samples in the 2D DCV feature space for five subjects from the CMU-PIE database.

Figure 4 shows that the distribution of the synthesized images (Figure 3b) is similar to that of the real images (Figure 3a). In both subfigures (Figure 3a,b), the samples are favorably clustered according to each subject, and there is less overlap between samples for the different subjects. Meanwhile, in the distributions resulting from other methods, some of the samples belonging to the same subject are widely distributed or overlap with those of other subjects. Although the samples obtained by BIF (Figure 4d) show favorably distinguished clusters, BIF is limited when expressing diverse changes in images owing to a limited number of synthesizable images (approximately seven images); it also requires front-lighted images to generate new images. In contrast, the proposed method can generate considerably more illumination conditions, resulting in its effectiveness in dealing with possible changes in the actual illumination environment. It can also produce images containing diverse illumination conditions using a single image captured under an arbitrary illumination condition.

## 3. Experimental Results

### 3.1. Image Generation Results

We tested the proposed relighting method for well-known face databases. As mentioned earlier, the coupled bilinear model was constructed based on 500 3D shape–image pairs from the FRGC 2.0 database [51]. We generated 100 illumination images for each 3D sample, which were used to train the proposed model. The sizes of the core tensors were 40×30×10×150 for the image model and 40×30×100×150 for the illumination model. The parameters for the proposed method were set as λ=0.3 and γ=0.8. First, we performed 10-fold cross-validation on the 500 samples of the FRGC 2.0 database to confirm the processing speed and errors. Synthesizing 100 relit images from a single input took 0.24 seconds on average (in MATLAB), and the root mean square (RMS) errors of the relit images (from the ground truth images) was 0.086 on average.

**Figure 5:**
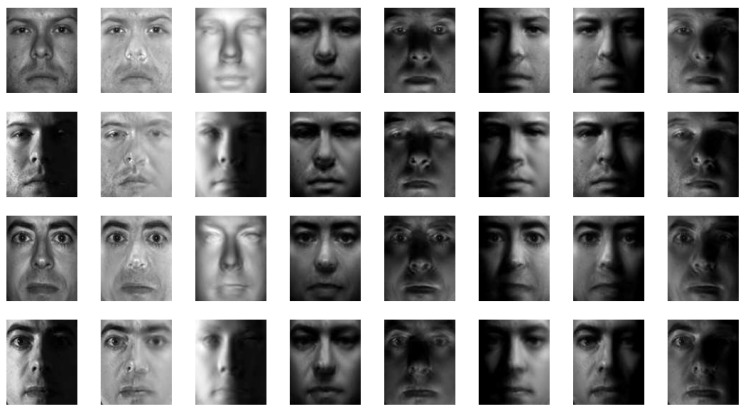
Relit images based on the Yale database (1st column: input image; 2nd: recovered texture; 3rd: estimated illuminance; and 4–8th: relit images).

**Figure 6:**
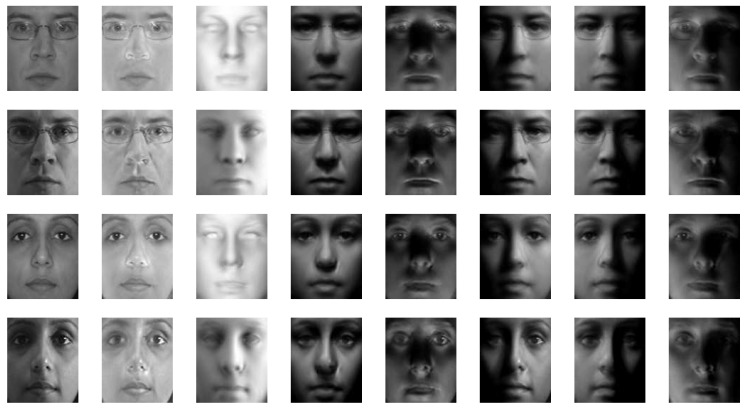
Relit images based on the Multi-PIE database (1st column: input image; 2nd: recovered texture; 3rd: estimated illuminance; and 4–8th: relit images).

The proposed method was applied to the Yale [55] and Multi-PIE [56] databases for qualitative evaluations. Figure 5 and Figure 6 show some of the reconstruction results. Here, we can see that the method can generate relit images robustly, irrespective of the input light condition, and it can fill areas under heavy shadows. Moreover, the results are unaffected by small outliers, such as the glasses in the examples. Textures are robustly recovered without degrading the resolution, which makes the relit images more realistic, even though the method does not involve any 3D reconstruction procedures.

**Figure 7:**
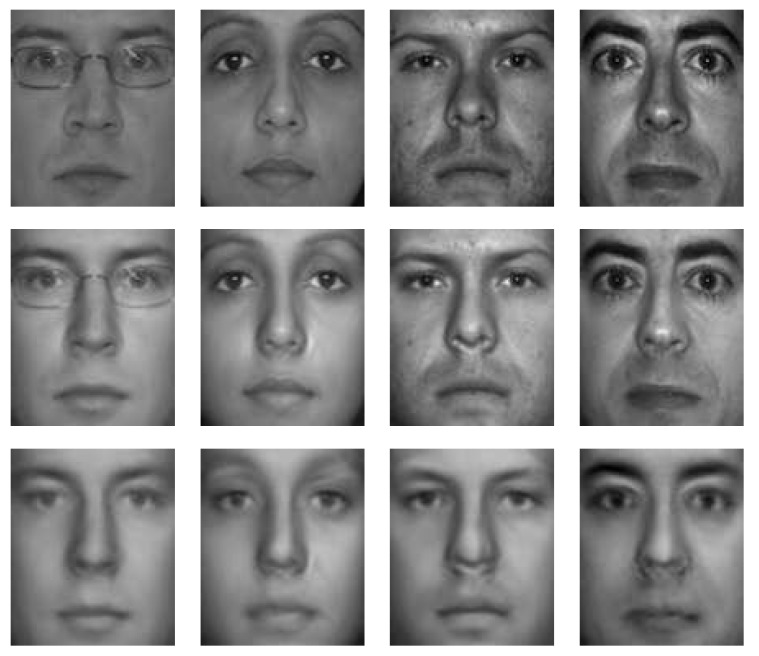
Comparison between the coupled bilinear model and the single image model (1st row: input image; 2nd: coupled bilinear model; and 3rd: image model only).

To confirm whether the coupled model can indeed provide better-quality images than the virtual images generated by a single subspace model, we also synthesized relit images using only the image model (Equation (4)) as was performed in the early subspace-based approaches [40,41,42,43]. Figure 7 compares the results of the coupled model and the single image model for the same inputs. From this, we can confirm that the images from the coupled model are better quality. The results from the single image model are poor and blurry, unlike those of the proposed method.

### 3.2. Performance of Face Recognition in SSPP Problems

To build a face recognition system that can robustly handle diverse changes in the actual environment, we synthesized images under several illumination conditions based on a single image by using the proposed method, and we constructed a feature space for face recognition, together with the synthesized images. In order to confirm the effectiveness of the proposed method, we compared the face recognition performance of the proposed method with those of other methods (E(${\mathrm{PC}}^{2}$)A2+, SPCA+, BIF, SLC, SRC, ESRC, RADL [29]), which were developed to solve the SSPP problem. For BIF, 7 images of 40 subjects contained in the CMU-PIE database [57] were used to synthesize the virtual images given in [13].

For feature extraction, we employed DCV [9] in the experiments; the one-nearest-neighbor rule was employed as a classifier, and the Euclidean distance was used as a measurement between samples [58,59].

**Figure 8:**
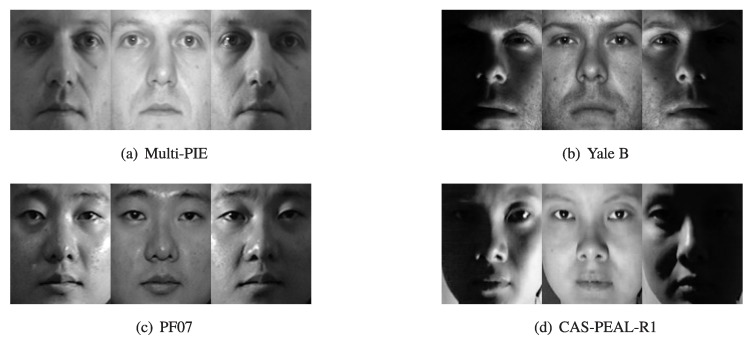
Sample images of each database: (**a**) Multi-PIE; (**b**) Yale B; (**c**) PF07; and (**d**) CAS-PEAL-R1.

The face recognition performance was evaluated by the recognition rates for the images in the Multi-PIE, Yale B [60,61], Postech Face07(PF07) [62], and CAS-PEAL-R1 databases [63]. The characteristics and the sample images from each database are presented in Table 1 and Figure 8, respectively.

For face alignment, the center of each eye was manually detected in all of the images, and the subsequent horizontal alignment of the eyes was achieved with rotation, as given in [7,12]. All the images were cropped and rescaled to ensure that the central point of each eye was statically positioned in an image with a size of 120 × 100 pixels. Then, the histogram equalization process [6] was applied to the rescaled image.

The Multi-PIE database contains over 75,000 images captured from 337 subjects, who were recorded in up to four sessions over a span of 5 months. The number of subjects participating in each session varied from a minimum of 203 to a maximum of 249. The images of each subject comprise different viewpoints, illumination conditions, and facial expressions. Among them, images of 249 subjects with a neutral expression captured from frontal viewpoints with 20 different illumination conditions were used in this experiment. Images with an illumination index of ‘8’ for each subject, which were captured with the frontal lighting condition, were used as input for virtual image generation, and the face recognition rates were evaluated for the remaining 19 images (total of 4731 images = 249 subjects × 19 images).

The Yale B database contains images from 10 subjects, each one captured in 64 images with different illumination conditions, and the Extension Yale B database provides images of an additional 28 subjects. The Yale B database categorizes the images into five subsets (indexed 1, 2, 3, 4, and 5) according to the lighting angle from the frontal view; a larger index denotes an angle of lighting that is more distant from the frontal view. For each subject, one image with frontal illumination was selected for virtual image generation, and the remaining 63 images (a total of 2394 images = 38 subjects × 63 images) were used to test the recognition performance.

The PF07 database contains a total of 60,000 images captured from every subject (200 subjects). In this study, we used a total of 3200 frontal inexpressive images of 200 subjects under 16 different illumination conditions. The CAS-PEAL-R1 database comprises images captured under 15 different illumination conditions from 8 subjects. Likewise, one image was selected among them to generate virtual images, and the remaining images were used for the face recognition test.

For the experiments on each database, the BIF method generated six virtual images with different illuminations for each subject. E(${\mathrm{PC}}^{2}$)A2+ generated three images for training from the original image, which correspond to the half-, first-, and second-order projected images, respectively. In SPCA+, 7 images (which were obtained from different n-order singular values) for each subject were generated for training, and, in SLC, 11 images for each subject were added to the training set—these were symmetric images and linear combination virtual images. The auxiliary intraclass variant dictionary for ESRC was generated with the images used for training BIF [13]. Note that the number of images and parameters of these compared methods were selected based on their original paper.

**Figure 9:**
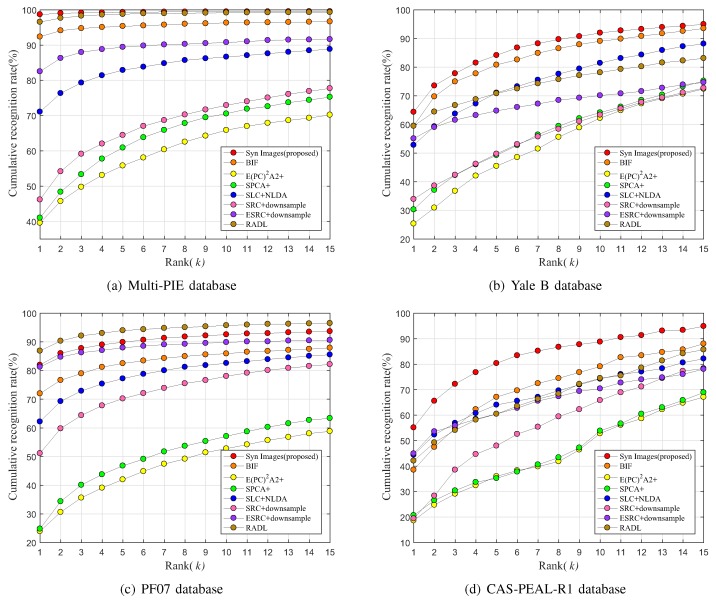
Face recognition rates for various databases: (**a**) Multi-PIE; (**b**) Yale B; (**c**) PF07; and (**d**) CAS-PEAL-R1.

For each database, the face recognition performance was evaluated using the cumulative match score [64]. For a given probe image $p_{i}$, the target images $t_{k}$ (k=1,…,15) are sorted by their similarity scores $s_{i}\left(\cdot \right)$ based on the Euclidean distance. Figure 9 shows the experimental results, where the horizontal axis represents the rank *k* and the vertical axis corresponds to the cumulative recognition rate of the top *k* matches. In Figure 9, the face recognition performance of the proposed method appears to be mostly superior to other methods at each database with respect to all features, regardless of the number of features. For the cases of E(${\mathrm{PC}}^{2}$)A2+ and SPCA, face recognition in the presence of actual environmental change was somewhat ineffective because the images were generated through SVD perturbation, projection, or linear combination, with no consideration for the variation in the actual face images caused by environmental changes. Although the images generated by BIF reflect the changes in illumination conditions, BIF is limited in the number of images that can be created. On the other hand, the proposed method reflects the probable image changes from the actual environment quite effectively in the generated image and, consequently, results in better face recognition performance than that of BIF. RADL shows worse performance than the proposed method, with the exception of experiments on the PF07 database; in particular, for the Yale B and the CAS-PEAL-R1 databases, RADL’s performance is far worse than the best performance achieved by the other methods. Note that the Multi-PIE and PF07 databases contain only horizontal variations of illuminations, such as the CMU-PIE database used for training all the face recognition methods, unlike the Yale B and the CAS-PEAL-R1 databases, which also contain vertical variations. Although the RADL method shows a slightly higher recognition rate than the proposed method for the PF07 database, the difference is not significant, and both the proposed method and the RADL method demonstrate good performances. However, for Yale B and CAS-PEAL-R1, which have more varied illumination changes, RADL is not as good as the other algorithms. This suggests that the proposed method has a better generalization performance.

The results of face recognition shown in Figure 9 appear consistent with those in Figure 4. The methods (the proposed method, BIF, and SLC) which show favorable clusters of the synthesized images by each subject in Figure 4a,d,e have higher face recognition rates than the other methods (E(${\mathrm{PC}}^{2}$)A2+, SPCA+) in Figure 4b,c. For SLC, although it achieves clusters of low variance in Figure 4e, it has a lower face recognition performance for the other databases, except CAS-PEAL-R1, than those of BIF and the proposed method because it is unable to properly reflect the actual changes in the image of one person.

### 3.3. Data Augmentation in Deep Learning

The proposed virtual image synthesis method can also be used in deep learning for data augmentation. In order to demonstrate the use of the proposed method in deep learning, SphereFace [65]—a deep network for face recognition—was trained with augmented data sets based on the synthesis methods used in several of the previous experiments: the proposed method, BIF, E(${\mathrm{PC}}^{2}$)A2+, SLC, and SPCA+. Note that SRC, ESRC, and RADL were not evaluated here because they are not based on virtual image generation. The original paper presenting SphereFace [65] describes two protocols for face recognition, so we performed experiments similarly:

- **Closed set:** In this case, the classes of the training and test data are identical. The classification result of each sample is derived from the softmax operator of the network. For the closed-set experiment, we picked one image with frontal illumination from each class and generated virtual images to construct the training set. The rest of the images in the DB composed the test set. The network was trained or fine-tuned based on the training set, and the recognition rate was evaluated based on the softmax results of the test set.
- **Open set:** This is the case wherein the classes in the training and test data are mutually exclusive. The classification result of each test sample is determined based on the cosine distances from gallery samples, where the cosine distance is evaluated based on the features of the final network layer. For this experiment, we divided the DB into two equally sized sets with disjoint classes. One set was used for training or fine-tuning (the training samples were generated in a manner similar to those for the closed set experiment). The other set was used to generate gallery and test samples (i.e., one sample for each class was picked); virtual images were generated to compose the gallery set, and the rest of the samples were used as test samples. Performance was evaluated similarly to the closed-set experiment, except that decisions were made based on the cosine distances from the gallery samples.

For both cases, we either fine-tuned the network based on a pre-trained model generated by the authors, or we trained the network from scratch.

Table 2, Table 3, Table 4 and Table 5 show the performance results of these experiments. Here, we can see that the proposed method provides the best result most of the time; in the one exception, the proposed method is the second best. These results show that the proposed virtual image synthesis method is also effective for augmenting data for deep learning.

## 4. Conclusions

Ideally, to establish a face recognition system which is robust to a variety of environmental changes, those changes should be included in the training procedure. However, the task of securing many images of each person for the pre-training is time-consuming and costly. This is one of the substantial challenges in the buildup of a practical face recognition system. We propose an image generation method to solve the SSPP problem, and we designed a face recognition system which is robust to such environmental changes as illumination. The proposed method, based on a carefully designed coupled bilinear model, synthesizes numerous realistic virtual images under various light conditions in a short time. Inspired by the knowledge that illuminance is less sensitive to the poor quality of a subspace-based model than reflectance (or texture) and that facial illuminance has a strong correlation to the input image, we designed a coupled model that efficiently extracts the illuminance from the input image; such a process would normally require a complicated 3D fitting procedure. The proposed method then estimates the texture information based on the extracted illuminance and the input image without negatively affecting the high-frequency components, and this operation is robust to the presence of heavy shadows. Of the 100 images synthesized from a single image captured under arbitrary light conditions, a portion was selected to construct the feature space by accounting for the distributions of the real images captured under diverse illumination conditions. Through the experiments, the distributions of the selected synthesized images in the feature space were found to be similar to those of real images, and the proposed method achieved better face recognition performance than the other SSPP methods using various databases. The proposed image synthesis method is relatively simple and concise, so it can be easily adapted to other larger face recognition frameworks, such as [32], or it can be used as a data augmentation scheme in recently popular deep-learning-based methods. There are factors other than illumination, such as pose, occlusion, makeup, and facial variation, to consider for the SSPP problem. Attempting to manage many of these factors at once efficiently is a challenging task and is left as future work.

## Figures and Tables

**Table 1:** Characteristics of each database.

	Multi-PIE	Yale B	PF07	CAS-PEAL-R1
No. of subject	249	38	200	28
No. of images per subject	20	64	16	15
Illumination variation	large	large	large	large
Expression variation	none	none	none	none

**Table 2:** Face recognition rate of SphereFace [65] on CAS-PEAL-R1 with various data augmentation methods.

Algorithm	Closed Set		Open Set	
	Fine-Tuning	from Scratch	Fine-Tuning	from Scratch
Proposed				
hltextbf38.27%	**32.14%**	**48.47%**	**31.12%**	
BIF	30.36%	11.99%	46.94%	13.27%
E(PC)${}^{2}$A2+	26.79%	9.18%	44.39%	9.69%
SLC	29.85%	19.90%	37.76%	10.20%
SPCA+	25.77%	7.40%	43.37%	9.18%

**Table 3:** Face recognition rate of SphereFace [65] on Multi-PIE with various data augmentation methods.

Algorithm	Closed Set		Open Set	
	Fine-Tuning	from Scratch	Fine-Tuning	from Scratch
Proposed	**89.24%**	**85.71%**	84.67%	**91.92%**
BIF	65.00%	44.45%	73.35%	63.62%
E(PC)${}^{2}$A2+	63.71%	49.27%	71.37%	48.13%
SLC	57.37%	30.56%	54.27%	28.04%
SPCA+	72.10%	58.19%	**88.21%**	71.07%

**Table 4:** Face recognition rate of SphereFace [65] on PF07 with various data augmentation methods.

Algorithm	Closed Set		Open Set	
	Fine-Tuning	from Scratch	Fine-Tuning	from Scratch
Proposed	**75.40%**	**59.67%**	**69.67%**	**52.27%**
BIF	51.77%	32.37%	67.20%	34.00%
E(PC)${}^{2}$A2+	42.57%	18.40%	54.73%	29.27%
SLC	49.53%	25.53%	53.47%	33.73%
SPCA+	35.77%	39.70%	68.27%	41.13%

**Table 5:** Face recognition rate of SphereFace [65] on Yale B with various data augmentation methods.

Algorithm	Closed Set		Open Set	
	Fine-Tuning	from Scratch	Fine-Tuning	from Scratch
Proposed	**63.32%**	**51.13%**	**62.49%**	**41.27%**
BIF	47.87%	38.76%	55.72%	34.25%
E(PC)${}^{2}$A2+	41.10%	13.24%	54.55%	20.22%
SLC	40.43%	30.74%	51.63%	27.40%
SPCA+	46.95%	35.51%	54.39%	21.22%
